# Increased Hippocampus–Medial Prefrontal Cortex Resting-State Functional Connectivity and Memory Function after Tai Chi Chuan Practice in Elder Adults

**DOI:** 10.3389/fnagi.2016.00025

**Published:** 2016-02-16

**Authors:** Jing Tao, Jiao Liu, Natalia Egorova, Xiangli Chen, Sharon Sun, Xiehua Xue, Jia Huang, Guohua Zheng, Qin Wang, Lidian Chen, Jian Kong

**Affiliations:** ^1^College of Rehabilitation Medicine, Fujian University of Traditional Chinese Medicine, Fuzhou, China; ^2^Fujian Key Laboratory of Rehabilitation of Technology, Fuzhou, China; ^3^Department of Psychiatry, Massachusetts General Hospital, Harvard Medical School, Boston, MA, USA; ^4^The School of Social and Political Science, University of Edinburgh, Edinburgh, UK; ^5^Affiliated Rehabilitation Hospital, Fujian University of Traditional Chinese Medicine, Fuzhou, China

**Keywords:** Tai Chi Chuan exercise, Baduanjin exercise, memory function, aging, hippocampus, medial prefrontal cortex

## Abstract

Previous studies provide evidence that aging is associated with the decline of memory function and alterations in the hippocampal (HPC) function, including functional connectivity to the medial prefrontal cortex (mPFC). In this study, we investigated if longitudinal (12-week) Tai Chi Chuan and Baduanjin practice can improve memory function and modulate HPC resting-state functional connectivity (rs-FC). Memory function measurements and resting-state functional magnetic resonance imaging (rs-fMRI) were applied at the beginning and the end of the experiment. The results showed that (1) the memory quotient (MQ) measured by the Wechsler Memory Scale-Chinese Revision significantly increased after Tai Chi Chuan and Baduanjin practice as compared with the control group, and no significant difference was observed in MQ between the Tai Chi Chuan and Baduanjin groups; (2) rs-FC between the bilateral hippocampus and mPFC significantly increased in the Tai Chi Chuan group compared to the control group (also in the Baduanjin group compared to the control group, albeit at a lower threshold), and no significant difference between the Tai Chi Chuan and Baduanjin groups was observed; (3) rs-FC increases between the bilateral hippocampus and mPFC were significantly associated with corresponding memory function improvement across all subjects. Similar results were observed using the left or right hippocampus as seeds. Our results suggest that both Tai Chi Chuan and Baduanjin may be effective exercises to prevent memory decline during aging.

## Introduction

Age-related cognitive impairment is a burgeoning public health concern throughout the world. Studies have suggested that while activities of daily living generally remain intact, about one in four older adults will experience a decline in a specific cognitive domain: memory (Unverzagt et al., 2001).

Previous studies have suggested that the hippocampus is the key region in memory function (Burgess et al., 2002; Kumaran and Maguire, 2005; Bird and Burgess, 2008; Roche et al., 2009; Kim et al., 2015; Mattfeld and Stark, 2015). Age-related memory changes in hippocampal (HPC) function and connectivity have long been the subject of research (Miller et al., 2008; Roche et al., 2009; Salami et al., 2014).

Another brain region that is believed to play an important role in memory is the medial prefrontal cortex (mPFC). Studies have suggested that the mPFC is involved in consolidation of memory, while the hippocampus is involved in retrieval of formed memories (Takehara-Nishiuchi and McNaughton, 2008). More recently, accumulating evidence has shown that HPC–medial prefrontal (MPFC) interactions may play a crucial role in the assimilation of new memories into pre-existing networks of knowledge and modulate the consolidation process of turning new memories into a permanent storehouse of knowledge (van Kesteren et al., 2012, 2013; Brod et al., 2013; Preston and Eichenbaum, 2013).

Studies showed that physical activity or mental training practices could slow the progression of cognitive and neural decline in healthy older adults (Burdette et al., 2010; Hayes et al., 2013; Voss et al., 2013; Wells et al., 2013; Kelly et al., 2014). In addition, investigators also found that meditation can also modulate the structure and function of the medial temporal lobe, including the hippocampus (Hölzel et al., 2008; Luders et al., 2009; Wells et al., 2013).

Tai Chi Chuan and Baduanjin are two common forms of mind–body exercises, which originated in China as martial arts (Wang et al., 2010; Zheng et al., 2014) and combine slow movements and deep breathing to facilitate smooth vital energy (qi) flow in the body (Wang et al., 2010). Recent literature has demonstrated their important role in disease treatment and prevention, as well as health maintenance (Wang et al., 2010; Li et al., 2012; Mei et al., 2012; Lan et al., 2013; Manson et al., 2013; Black et al., 2014; Taylor-Piliae et al., 2014; Cheng, 2015; Xiong et al., 2015).

Both Tai Chi Chuan and Baduanjin are complex interventions, including physical, emotional, spiritual, and psychosocial components. Despite the similarities, they each have their own characteristics. Compared to Baduanjin, which involves eight simple fixed movements of arms with almost no movement of legs (Xiong et al., 2015), Tai Chi Chuan is much more complex and requires moving the body and four limbs by spatial navigation toward oneself (Wei et al., 2013). As a result, the two mind–body practices may target different populations. For those in good physical condition, Tai Chi Chuan may be a good choice; for those in poor physical condition, particularly having problems with their legs or suffering from memory impairment, so that they cannot learn the complicated procedures of Tai Chi Chuan, Baduanjin may be a better option.

Accumulating evidence suggests that Tai Chi Chuan and Baduanjin practices can improve cognitive performance (Wang, 2007; Lam et al., 2011; Tsai et al., 2013; Fong et al., 2014; Li et al., 2014a; Wayne et al., 2014; Yin et al., 2014). For instance, Mortimer et al. (2012) found that with 40 weeks of practice (three times per week), Tai Chi Chuan could improve Mattis Dementia Rating Scale scores, including memory score, compared to the no intervention control. Man et al. (2010) found that Tai Chi Chuan can improve memory function as measured by the Rivermead Behavioral Memory Test and the Hong Kong List Learning Test compared to regular exercise and non-exercise controls.

In this study, we investigated how longitudinal Tai Chi Chuan and Baduanjin can modulate memory function and HPC resting-state functional connectivity (rs-FC) in elderly adults. Given the important role of the communication between the hippocampus and mPFC in memory processes (Churchwell and Kesner, 2011; Hyman et al., 2011; van Kesteren et al., 2012; Brod et al., 2013; Preston and Eichenbaum, 2013; Bein et al., 2014; Kaplan et al., 2014; Griffin, 2015; Kurczek et al., 2015), we hypothesized that both Tai Chi Chuan and Baduanjin practices may improve memory function by increasing HPC functional connectivity with the mPFC.

## Materials and Methods

### Participants

The Medical Ethics Committee in the Affiliated Rehabilitation Hospital of Fujian University of Traditional Chinese Medicine approved all study procedures. The experiment was performed in accordance with approved guidelines. All participants signed a written consent. This study was registered on the Chinese Clinical Trial Registry (ChiCTR)^1^ (ChiCTR-IPR-15006131).

We recruited healthy older volunteers aged 50–70 in one community (Sports Center Community) in Gulou District, Fuzhou City, China. Two cohorts of elderly adults were recruited independently in the same community to avoid potential cross-practice between Tai Chi Chuan and Baduanjin. Subjects were randomized to the Tai Chi Chuan or control group in one cohort and to the Baduanjin or control group in the other cohort. The two cohorts started and ended at the same time.

Inclusion criteria for study participants were aged between 50 and 70 years; no regular physical exercise for at least 1 year (3 months with a frequency of three to four times per week and 30 min/session were considered the minimal standard for regular physical exercise); right-handedness; ability to provide written informed consent. Subjects were excluded from the study for any of the following: history of stroke; any severe cerebrovascular disease, musculoskeletal system diseases, or other sports injury-related contraindications; cognitive screening by the Mini-Mental State Exam (MMSE) <24 (Folstein et al., 1975); and Beck depression inventory (BDI) ≥14.

**Figure 1:**
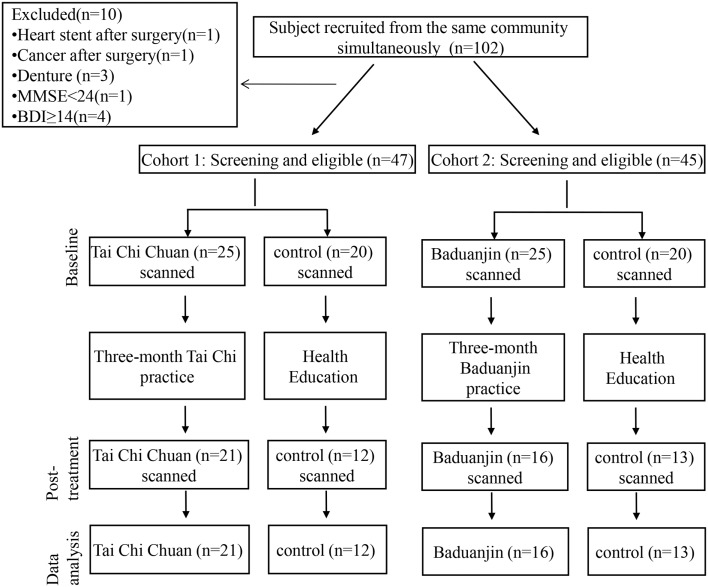
**Flow diagram for the screening, randomization, and completion of 12-week evaluations**.

Of the 90 subjects who passed screening and finished baseline scans in this study, 62 healthy older volunteers (21 in the Tai Chi Chuan group, 16 in the Baduanjin group, and 25 in the control group) completed all study procedures and fMRI scans (Figure 1). Four subjects did not complete the study in the Tai Chi Chuan group due to schedule conflicts (2), dwelling relocation (1), or unwillingness to receive the second MRI scan (1). Nine subjects did not complete the study in the Baduanjin group due to schedule conflicts (8) or unwillingness to participate in the second MRI scan (1). Of the 15 subjects who dropped out in the control group, 11 were due to scheduling conflicts and 4 due to unwillingness to participate in the second MRI scan.

### Intervention

#### Tai Chi Chuan Exercise Group

The Tai Chi Chuan exercise took place 5 days/week for 12 weeks with each session lasting 60 min. It was based on Yang-style 24-form (China National Sports Commission, 1983), which is recommended as a popular health activity by the General Administration of Sport in China. Each session included a sequence of 10 min of warm-up and review of Tai Chi Chuan principles, 30 min of Tai Chi Chuan exercises, 10 min of breathing techniques, and 10 min of relaxation.

#### Baduanjin Exercise Group

The Baduanjin exercise was based on “Health Qigong – Baduanjin” recommendations published by the General Administration of Sport in China (Health Qigong Management Center of General Administration of Sport of China, 2003). It was conducted 5 days/week for 12 weeks, for 60 min/session. The whole set of Baduanjin contains 10 postures, including the starting and ending postures. The time schedule of the Baduanjin group was the same as that of the Tai Chi Chuan group. Each session included a warm-up followed by a review of principles, movements, breathing techniques, and relaxation.

Tai Chi Chuan and Baduanjin classes were taught by two professional instructors from the Fujian University of Traditional Chinese Medicine with more than 5 years of training experience. In addition, the training procedure was supervised by two staff members to guarantee the quality of the research.

#### Control Group

Participants in the control group received basic health education at the beginning of the experiment. During the following 12-week period, subjects were asked to maintain their original physical activity habits. Free Tai Chi Chuan or Baduanjin training was offered to them after the research period.

### Memory Function Measurement

We measured memory function using the Wechsler Memory Scale-Chinese Revision (WMS-CR) (Woodard and Axelrod, 1987; Gong and Wang, 1989), which is composed of 10 subtests (information, orientation, mental control, picture, recognition, visual reproduction, associative learning, touch, comprehension memory, and digit span) and an overall memory quotient (MQ). It is designed for the assessment of memory function and is frequently used for clinical assessment procedures. The measurement was performed by two blinded licensed WMS-CR raters at the beginning and end of the study. WMS-CR applied at the beginning and the end of the study used the same materials.

### fMRI Data Acquisition

Each subject participated in two identical fMRI scanning sessions at the beginning and the end of the study. fMRI data were acquired on a 3.0-T GE scanner (General Electric, Milwaukee, WI, USA) with an eight-channel phased-array head coil. Subjects were asked to stay awake and remain motionless during the scan with their eyes closed and ears plugged. Prior to the functional run, magnetization-prepared rapid gradient echo (MPRAGE) T1-weighted images were collected with the following parameters: flip angle = 15°, 1 mm slice thickness, 240 mm field of view (FOV), and 164 images (slices) in acquisition. Resting-state fMRI, data were acquired with TR = 2100 ms, TE = 30 ms, flip angle = 90°, slice thickness = 3 mm, gap = 0.6 mm, acquisition matrix = 64 × 64, voxel size = 3.125 mm × 3.125 mm × 3.6 mm, 42 axial slices, FOV = 200 mm × 200 mm, phases/location = 160. Each scan lasted 5 min 36 s.

### Statistical Analysis

#### Behavioral Data Analysis

Behavioral analysis was performed using SPSS 18.0 Software (SPSS Inc., Chicago, IL, USA). One-way ANOVA and Chi-square tests were applied to compare the baseline characteristics of the subjects between groups. For this analysis, control subjects from two cohorts were combined in one group to increase the power. We performed two-sample *t*-tests to make sure that there were no differences between control subjects drawn from the two cohorts (Tai Chi Chuan control *n* = 12, Baduanjin control *n* = 13). The result showed there is no significant differences between the two control groups in age (*p* = 0.928), gender (*p* = 0.409), years of education (*p* = 0.151), as well as baseline MQ (*p* = 0.593) and MQ improvement (post − pre) (*p* = 0.671). To estimate the effects of Tai Chi Chuan and Baduanjin, we compared MQ scores pre- and posttreatment using a mixed-model regression with subjects as a random effect, group (Tai Chi Chuan, Baduanjin, and control), time point (week 0 and 12), age, gender, and years of education as fixed effects.

#### Resting-State fMRI: Seed-to-Voxel Analysis

**Figure 2:**
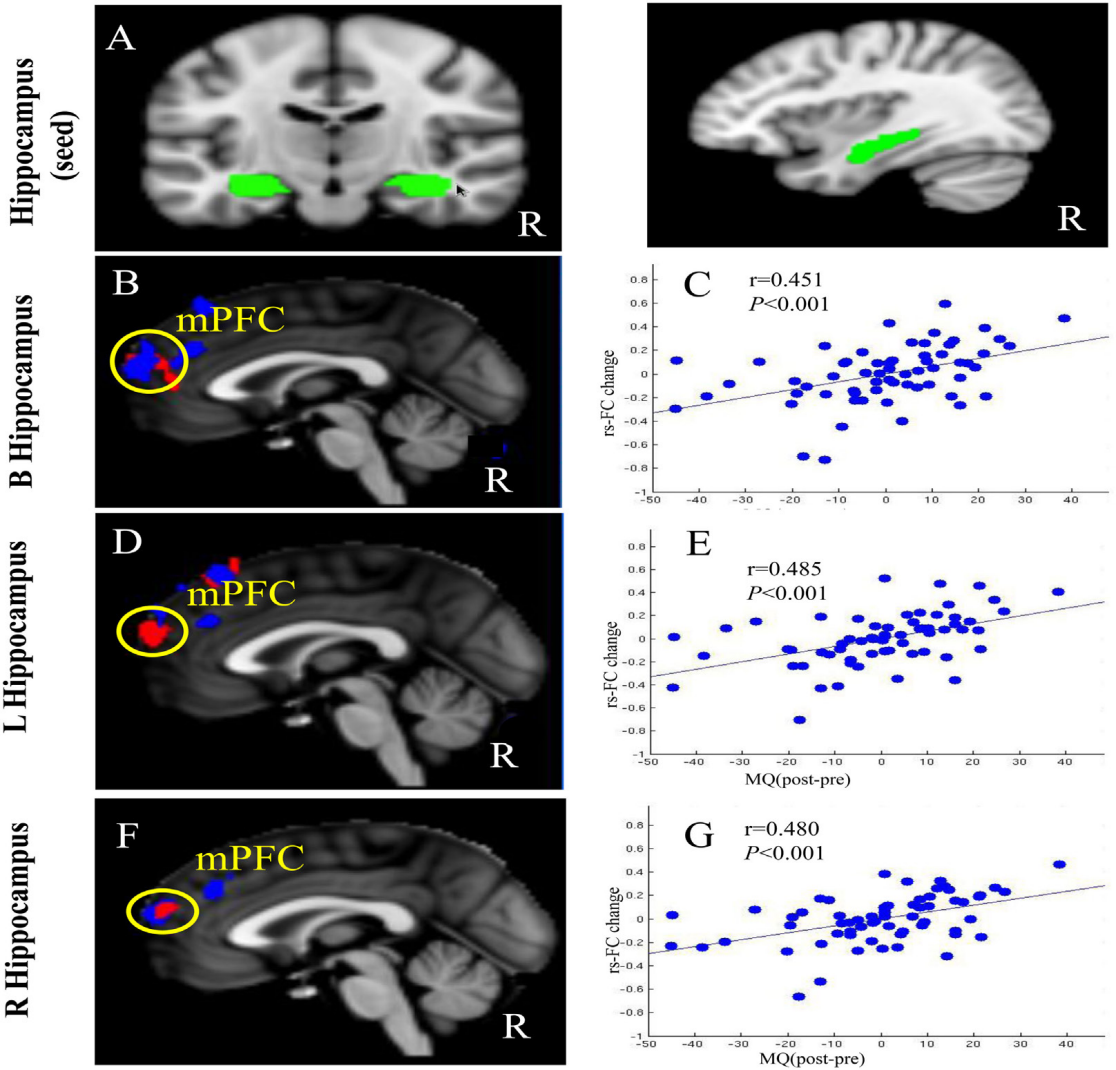
**(A)** Bilateral, left, and right hippocampus as seeds. **(B,D,F)** Significant resting-state functional connectivity changes in the Tai Chi Chuan group compared to the control group (red) and the brain regions that showed significant association between the connectivity increase and memory improvement (blue), overlapping in the mPFC. **(C,E,G)** Scatter plots indicate the correlation between memory quotient (MQ) and Fisher’s *Z* values at the peak of the significant cluster, corrected for age, gender, and years of education. B, bilateral; R, right; L, left.

Functional connectivity analysis was carried out by applying a seed-based approach using the CONN toolbox v14.p (Whitfield-Gabrieli and Nieto-Castanon, 2012).^2^ Left, right, and bilateral hippocampus templates extracted from the AAL (Tzourio-Mazoyer et al., 2002) using WFU-Pick Atlas software (Maldjian et al., 2003) were selected as seeds (Figure 2A).

The preprocessing of fMRI data was performed using Statistical Parametric Mapping (SPM8) (Wellcome Department of Cognitive Neurology, University College, London, UK) in MATLAB (Mathworks, Inc., Natick, MA, USA). The preprocessing steps included slice-timing, realignment, coregistration to subjects’ respective structural images, normalization, and smoothing with an 8-mm full width at half maximum (FWHM) kernel. In addition to these steps, we employed segmentation of gray matter, white matter, and cerebrospinal fluid (CSF) areas for the removal of temporal confounding factors (Whitfield-Gabrieli and Nieto-Castanon, 2012). Band-pass filtering was performed with a frequency window of 0.01–0.1 Hz.

To eliminate correlations caused by head motion and artifacts, we identified outlier time points in the motion parameters and global signal intensity using ART.^3^ For each subject, we treated images as outliers if composite movement from a preceding image exceeded 0.5 mm, or if the global mean intensity was >3 SDs from the mean image intensity for the entire resting scan. Outliers were included as regressors in the first-level general linear model along with motion parameters. First-level correlation maps were produced by extracting the residual BOLD time course from each HPC seed and by computing Pearson’s correlation coefficients between that time course and the time courses of all other voxels in the brain. Correlation coefficients were Fisher transformed into “*Z*” scores, which increases normality and allows for improved second-level General Linear Model analyses.

Whole brain group analysis was applied using two-sample *t*-tests to compare the hippocampus functional connectivity changes between different groups. To investigate the association between the functional connectivity change and the corresponding MQ changes, we also performed a whole brain regression analysis between the change in MQ (post − pretreatment) and the corresponding changes in the hippocampus functional connectivity (post − pretreatment) across all the subjects. Age, gender, and years of education were included in the analysis as covariates of non-interest.

A threshold of voxel-wise *p* < 0.005 uncorrected and cluster-level *p* < 0.05 family wise error (FEW) correction was applied for all fMRI data analysis.

## Results

### Clinical Outcomes

Demographic characteristics for the 62 subjects who completed all study procedures are detailed in Table 1. There is no significant difference among the three groups in age, gender, handedness, and average years of education (*p* > 0.05). The average attendance rate in the Tai Chi Chuan group was 95%, ranging from 88 to 100%; in the Baduanjin group it was 97%, ranging from 92 to 100%.

**Table 1:** **Demographics of study participants in different groups**.

Characteristics*	Control (*n* **=** 25) Mean (SD)	Tai Chi Chuan (*n* **=** 21) Mean (SD)	Baduanjin (*n* **=** 16) Mean (SD)	*F*	**χ**^2^	*p*
Age^†^	59.76 (4.83)	62.38 (4.55)	62.18 (3.79)	2.386	–	0.101
Gender (female/male)^‡^	19/6	13/8	10/6	–	1.309	0.520
Handedness (right/left)	25/0	21/0	16/0	–
Average years of education^†^	8.52 (3.65)	9.61 (3.02)	9.06 (2.61)	0.671	–	0.515

*Control, control group; Tai Chi Chuan, Tai Chi Chuan group; Baduanjin, Baduanjin group*.

*All values are means (SD) and *p* < 0.05 for the difference between the groups unless otherwise noted.

*^†^One-way analysis of variance was calculated for age and average years of education*.

*^‡^Chi-square test was applied for gender comparison*.

The MQ pre- and posttreatment are shown in Table 2. At baseline measurement, there were no significant differences among the three groups. Mixed-model regression showed significant MQ increases in the Tai Chi Chuan and Baduanjin groups compared with the control group (Baduanjin: *p* < 0.0001, Tai Chi Chuan: *p* = 0.004). No significant differences were found between the Tai Chi Chuan and Baduanjin groups (*p* = 0.276).

**Table 2:** **Clinical outcome measurements in each group (pre- and posttreatment)**.

MQ^a^	Control (*n* **=** 25) Mean (SD)	Tai Chi Chuan (*n* **=** 21) Mean (SD)	Baduanjin (*n* **=** 16) Mean (SD)
Pretreatment	99.08 (14.59)	105.81 (10.24)	99.25 (8.99)
Posttreatment	97.76 (13.92)	123.57 (11.42)	125.06 (10.87)

*Control, control group; Tai Chi Chuan, Tai Chi Chuan group; Baduanjin, Baduanjin group*.

*^a^Significant difference on pre- and posttreatment changes using a mixed-model regression with subjects as a random effect and group (Tai Chi Chuan, Baduanjin, and control), time point (week 0 and 12), age, gender, and years of education as fixed effects*.

### Functional Connectivity Result

The results of the seed-to-voxel resting-state connectivity analysis are presented in Table 3 and Figure 2. After the 12-week practice, we found increased functional connectivity between the bilateral hippocampus and right mPFC and left mPFC in the Tai Chi Chuan group compared to the control group (Table 3; Figure 2B). Similar results were observed using the left and right hippocampus as seeds (Table 3; Figures 2D,F). There were no other contrasts that produced significant results.

**Table 3:** **Regions show significant functional connectivity changes with the hippocampus**.

Seed	Contrast	Brain regions	Cluster size (voxels)	Peak *z*-score	MNI coordinates (mm)		
					*X*	*Y*	*Z*
Bilateral hippocampus	Tai Chi > control	R mPFC	1327	3.50	12	58	16
		L mPFC		3.45	−12	52	16
L hippocampus	Tai Chi > control	R mPFC	695	3.78	16	48	14
		L mPFC		3.41	−12	52	16
R hippocampus	Tai Chi > control	R mPFC	641	2.96	12	58	16
		L mPFC		3.20	−12	58	16

*R, right; L, left; mPFC, medial prefrontal cortex*.

We did not find significant functional connectivity differences between the Baduanjin and control groups using the bilateral hippocampus as a seed at the initial threshold (*p* < 0.005, cluster-corrected at FWE *p* < 0.05) we set. As an exploratory analysis, we applied a relatively less conservative threshold of voxel-wise *p* < 0.05 and cluster-level *p* < 0.05 uncorrected and found greater connectivity between the bilateral hippocampus and the bilateral mPFC (MNI peak coordinate: 14, 32, 38; peak *Z* 3.34, voxels 3333). Similar results were observed in the Baduanjin group compared to the control group using the left and right hippocampus as seeds.

No significant functional connectivity differences between the Tai Chi Chuan and Baduanjin groups were observed using the bilateral, left, and right hippocampus as seeds.

Regression analyses between pre- and post-MQ change and the corresponding functional connectivity change in all subjects using the bilateral hippocampus as a seed showed a significant positive association at the bilateral mPFC (Table 4; Figure 2C). No negative association between the MQ change and the functional connectivity of the bilateral hippocampus was observed. Interestingly, we found that the connectivity results of the MQ regression analysis across all subjects and the Tai Chi Chuan versus control group comparison overlapped in the mPFC region (Table 4; Figure 2B). Similar results were found when using the right or left hippocampus as a seed (Table 4; Figures 2D–G).

**Table 4:** **Correlation between MQ and functional connectivity changes**.

Seed	Contrast	Brain regions	Cluster size (voxels)	Peak *z*-score	MNI coordinates (mm)		
					*X*	*Y*	*Z*
Bilateral hippocampus	Positive	R mPFC	1715	4.27	10	38	32
		L mPFC		3.80	−14	64	6
L hippocampus	Positive	R mPFC	675	4.00	12	32	40
		L mPFC		3.63	−14	64	6
R hippocampus	Positive	R mPFC	1073	4.17	10	38	32
		L mPFC		3.59	−16	38	40

*R, right; L, left; mPFC, medial prefrontal cortex*.

## Discussion

In this study, we investigated the memory performance and hippocampus rs-FC changes before and after 12 weeks of Tai Chi Chuan or Baduanjin practice compared with the control group in elderly adults. We found that MQ significantly increased in both Tai Chi Chuan and Baduanjin groups compared with the control group. Functional connectivity analysis using the bilateral, left, and right hippocampus as seeds showed that longitudinal Tai Chi Chuan practice significantly enhances hippocampus rs-FC with the mPFC. The modulation effect of the Baduanjin practice was weaker (significant at a less conservative threshold) but not significantly different from that of Tai Chi Chuan. Memory function improvement as indicated by the MQ score change was positively associated with rs-FC changes between the hippocampus and mPFC and overlapped with the observed group differences.

In this study, we found increased FC between the hippocampus and mPFC after Tai Chi Chuan practice. Previous studies suggest that the decline in memory performance that accompanies old age is associated with changes in both the hippocampus and the prefrontal cortex (Roche et al., 2009). Both animal and human studies suggest that the hippocampus is a critical brain region in memory function (Aggleton, 2014; MacDonald, 2014; Wixted et al., 2014). Studies also suggest that the mPFC is a major hub of the default mode network (Buckner et al., 2008) and is involved in integrating information from the external environment with stored internal representations (Miller, 2000). It controls top-down attention during conflict processing of alternative responses (Corbetta and Shulman, 2002) and is implicated in different aspects of social cognitive processing (Amodio and Frith, 2006; Van Overwalle, 2009).

Recently, investigators discovered that the mPFC plays an important role in memory processing (Macrae et al., 2004; Bañuelos et al., 2014) and undergoes changes with aging (Gutchess et al., 2007; Babakchanian et al., 2012; Van de Vijver et al., 2014). It has previously been shown that the hippocampus and mPFC make differential contributions to the neural network supporting introspection (Kurczek et al., 2015), the ability to remember the past and think about the future. The mPFC is mainly involved in the consolidation of memory while the medial temporal lobe including the hippocampus is mainly engaged in the retrieval of formed memory (Takehara-Nishiuchi and McNaughton, 2008). Studies showed that theta oscillations in the mPFC are modulated by spatial working memory and synchronize with the hippocampus through its ventral subregion (O’Neill et al., 2013). Other recent research findings suggested an increased need for “top-down” prefrontal control of HPC encoding processes to resolve the conflict between existing memories and new events as they are learned (Preston and Eichenbaum, 2013).

There is strong evidence supporting the communication between the hippocampus and mPFC, and its relevance for memory processes (Churchwell and Kesner, 2011; Hyman et al., 2011; van Kesteren et al., 2012; Brod et al., 2013; Preston and Eichenbaum, 2013; Bein et al., 2014; Kaplan et al., 2014; Griffin, 2015; Kurczek et al., 2015). For instance, van Kesteren et al. (2010, 2012, 2013) investigated how connections between the hippocampus and the vmPFC relate to the incorporation of new memories into existing abstract frameworks and found that HPC–vmPFC connectivity is enhanced during and shortly after successful encoding of novel information (van Kesteren et al., 2010).

In this study, we found that Tai Chi Chuan and Baduanjin can significantly improve memory performance. Both Tai Chi Chuan and Baduanjin are combined mind–body exercises, which consist of safe aerobic activities and mind training in sustained attention focusing and multi-tasking (Wayne et al., 2014). This result is consistent with previous studies that show aerobic exercise can significantly improve memory function (Flöel et al., 2010; Erickson et al., 2011).

We also found that the coupling of the hippocampus–mPFC connectivity increases after longitudinal Tai Chi Chuan practice and that the increased connectivity is associated with memory improvement. In a previous study, Li et al. (2014b) found that multimodal interventions including cognitive training, Tai Chi Chuan exercise, and group counseling can improve the resting-state connectivity between the mPFC and medial temporal lobe regions. But, as the author indicates, due to the multimodal versus control comparison, the study cannot clarify the contribution of each intervention component (cognitive training, Tai Chi Chuan, and group counseling). In another pilot study, Wells et al. (2013) found that longitudinal mindfulness-based stress reduction (MBSR) can increase functional connectivity between the posterior cingulate cortex and bilateral mPFC/left hippocampus compared to controls. Our result is consistent with these previous findings and in addition shows a comprehensive memory performance improvement after Tai Chi Chuan practice, as well as the connectivity increase between the mPFC and the hippocampus in the Tai Chi Chuan group compared to the controls. The association between memory improvement, hippocampus, and mPFC rs-FC in elderly individuals further endorses the anti-memory decline potential of Tai Chi Chuan practice.

With the Baduanjin practice, we found significant functional connectivity changes in the Baduanjin group compared to the control group only at a lower threshold than the Tai Chi Chuan versus control comparison. We speculate that this may be due to (1) a relatively smaller sample size in the Baduanjin group as compared to Tai Chi Chuan group (16 versus 21); and (2) Baduanjin is characterized by eight fixed movements, while Tai Chi Chuan is a much more complicated exercise. Given that no difference between the Tai Chi Chuan and Baduanjin groups was observed in a direct comparison, we speculate both Tai Chi Chuan and Baduanjin may improve memory function through improving the interaction between the hippocampus and mPFC. Further studies with a large sample size are needed to test this hypothesis.

In previous studies, Wei et al. (2013, 2014) investigated brain differences between highly experienced Tai Chi Chuan practitioners/masters and healthy controls (non-Tai Chi Chuan practitioners) to investigate whether brain differences existed between the two groups. They found that long-term Tai Chi Chuan practice could induce regional structural change and influence the intrinsic functional architecture. As a significant extension of these studies, we found that relatively short-term (3 months) Tai Chi Chuan practice can improve memory performance and resting-state FC in Tai Chi Chuan naive elderly adults, which suggests that individuals can benefit from Tai Chi Chuan practice in a relatively short time.

There are several potential limitations in this study. First, the sample size is relatively small. Second, both Tai Chi Chuan and Baduanjin are considered mind–body exercises. In this study, we could not tease apart the physical and mental components of the exercise and are therefore unable to conclude which component or combination of the two was crucial for memory improvement. Existing literature suggests that both body and mind exercises are important, and our study corroborates this claim. Future studies applying behavioral, brain imaging, and serum measurements to compare the effects of exercise, Yoga, meditation, Tai Chi Chuan, and Baduanjin are needed to elucidate specific effects of exercise and meditation in Tai Chi Chuan/Baduanjin.

In summary, we found that longitudinal intensive Tai Chi Chuan and Baduanjin practice can significantly improve comprehensive memory performance in elderly adults. Tai Chi Chuan practice can enhance rs-FC between the hippocampus and mPFC, and the increased connectivity is significantly associated with improvement of the memory function. Our result implies that Tai Chi Chuan and Baduanjin could be an efficient method for preventing memory decline during aging.

## Author Contributions

LC: experimental design; JK: analysis and manuscript preparation; JT: experimental design, data analysis, and manuscript preparation; GZ: data analysis; JL and XX: data collection and data analysis; JH, XC, and QW: data collection; SS and NE: manuscript preparation. All authors contributed to draft the manuscript and have read and approved the final manuscript.

## Conflict of Interest Statement

The authors declare that the research was conducted in the absence of any commercial or financial relationships that could be construed as a potential conflict of interest.

## Footnotes

^1^http://www.chictr.org.cn

^2^http://www.nitrc.org/projects/conn

^3^http://www.nitrc.org/projects/artifact_detect
